# *In Vivo* Depletion of Lymphotoxin-Alpha Expressing Lymphocytes Inhibits Xenogeneic Graft-versus-Host-Disease

**DOI:** 10.1371/journal.pone.0033106

**Published:** 2012-03-12

**Authors:** Eugene Y. Chiang, Ganesh Kolumam, Krista M. McCutcheon, Judy Young, Zhonghua Lin, Mercedesz Balazs, Jane L. Grogan

**Affiliations:** 1 Department of Immunology, Genentech Inc., South San Francisco, California, United States of America; 2 Department of Tumor Biology and Angiogenesis, Genentech Inc., South San Francisco, California, United States of America; 3 Department of Antibody Engineering, Genentech Inc., South San Francisco, California, United States of America; 4 Department of Assay and Automation Technology, Genentech Inc., South San Francisco, California, United States of America; New York University, United States of America

## Abstract

Graft-versus-host disease (GVHD) is a major barrier to successful allogeneic hematopoietic cell transplantation and is largely mediated by activated donor lymphocytes. Lymphotoxin (LT)-α is expressed by subsets of activated T and B cells, and studies in preclinical models demonstrated that targeted depletion of these cells with a mouse anti-LT-α monoclonal antibody (mAb) was efficacious in inhibiting inflammation and autoimmune disease. Here we demonstrate that LT-α is also upregulated on activated human donor lymphocytes in a xenogeneic model of GVHD and targeted depletion of these donor cells ameliorated GVHD. A depleting humanized anti-LT-α mAb, designated MLTA3698A, was generated that specifically binds to LT-α in both the soluble and membrane-bound forms, and elicits antibody-dependent cellular cytotoxicity (ADCC) activity *in vitro*. Using a human peripheral blood mononuclear cell transplanted SCID (Hu-SCID) mouse model of GVHD, the anti-human LT-α mAb specifically depleted activated LT-expressing human donor T and B cells, resulting in prolonged survival of the mice. A mutation in the Fc region, rendering the mAb incapable of mediating ADCC, abolished all *in vitro* and *in vivo* effects. These data support a role for using a depleting anti-LT-α antibody in treating immune diseases such as GVHD and autoimmune diseases.

## Introduction

Graft-versus-host disease (GVHD) is a complex immune disease underlying the morbidity and mortality associated with transplantation of hematopoietic stem cells into allogeneic recipients [1], [2], [3]. Induction of either acute or chronic GVHD occurs when transferred alloreactive donor T cells respond to antigens expressed on host tissues. The initial phase of acute GVHD development is mediated by the proinflammatory environment created by the tissue damage resulting from the conditioning regimen, including total body irradiation and chemotherapy. The release of proinflammatory cytokines, such as IL-1, IL-8 and TNF-α, triggers a cascade of inflammatory events including the activation and maturation of host antigen-presenting cells (APCs) that in turn present host major or minor histocompatibility antigen disparate proteins as complexes to donor T cells. These alloreactive T cells are the critical mediators of GVHD, secreting inflammatory cytokines (e.g. TNF-α, IFN-γ, IL-2) and cytolytic mediators, ultimately leading to the destruction of host organs, primarily the skin, GI tract and liver [2].

Chronic GVHD represents a multi-organ syndrome that shares many clinical manifestations with autoimmune diseases [4], [5]. While chronic GVHD is a major cause of morbidity and mortality in long-term survivors of allogeneic hematopoietic stem cell transplantation, the pathophysiology of chronic GVHD is poorly understood. As in acute GVHD, effector T cells and APCs play important roles. Additionally, B cells are also speculated to have a role, through direct cellular cytotoxicity by alloantibodies or as functional APCs capable of activating and expanding alloreactive T cells [5]. In chronic GVHD, alloantibody levels correlate with disease development [6], B cell-activating factor (BAFF) levels are high, and B cells with activated memory phenotype are present in greater numbers while naïve B cell numbers are reduced [7].

In clinical practice, standard first-line therapy against acute GVHD consists of corticosteroid treatment, as these agents are lympholytic and inhibit inflammatory cytokine cascades [8]. However, a significant patient population develops steroid-refractory/resistant GVHD that is associated with high morbidity and mortality [3], [8]. As primary response to first-line treatment is predictive of long-term survival, the lack of universally effective front-line therapy has driven the search for adjunctive therapies targeting the pathophysiological mechanisms involved in acute GVHD. Based on the roles of cellular effectors and soluble inflammatory mediators, biologics including monoclonal antibodies (mAbs) and fusion proteins have been evaluated as therapeutics against acute GVHD. Cell surface markers expressed by effector cells have been targeted with mAbs. These include CD2 (alefacept), CD3 (OKT3, visilizumab), CD25 (daclizumab, basiliximab, denileukin-difitox), CD52 (alemtuzumab), and CD147 (ABX-CBL). Strategies targeting cytokines include anti-TNF-α mAb (infliximab) and TNF receptor fusion proteins (etanercept) (reviewed in [3], [8]). While many of these strategies have shown at least some promising activity as salvage treatments in GVHD, due to the broad effects on the host immune system, patients are often still at risk for opportunistic infections or may develop lymphoproliferative disorders or reoccurrence of leukemia.

Therefore, more selective therapeutic strategies targeting activated pathogenic cells directly involved in GVHD may improve the net clinical benefit.

Lymphotoxin (LT)- α, is a TNF-superfamily member and exists as a soluble LT-α3 homotrimer that binds TNF receptors (TNFR), or complexed with LT-β as a heterotrimer, LT-α1β2, on the cell surface that binds to its cognate receptor LT-βR. The role of LT in the immune response has been well characterized and is crucial for the development and orchestration of robust immune responses [9]. Surface expression of LT-α is restricted to subsets of T and B cells. Activated CD4^+^ Th subsets Th1 and Th17, but not Th2, express surface LT [10], [11] as do CD8^+^ T cells and B cells [12], [13]. These cell types have all been implicated in the pathogenesis of GVHD. We recently demonstrated efficacy with a depleting anti-mouse LT-α mAb in mouse models of rheumatoid arthritis (RA), experimental autoimmune encephalomyelitis (EAE) and delayed-type hypersensitivity (DTH) [10]. In these studies, the Fc-dependent efficacy achieved with anti-LT-α treatment resulted in depletion of Th1 and Th17 cells, but not Th2.

The demonstrated efficacy of the depletion mechanism in inhibiting T cell-mediated diseases in mouse inflammatory models led us to look for mAbs directed against human LT-α with similar properties. We describe here that a humanized anti-LT-α mAb depleted activated T and B cells and increased survival in xenogeneic human T cell-dependent peripheral blood mononuclear cell (PBMC) transplanted SCID (Hu-SCID) mouse model of GVHD, whereas an Fc-effectorless mutant version of the antibody did not. These data support a role for using a depleting anti-LT-α antibody for eliminating pathogenic T and/or B cells in human inflammatory and autoimmune diseases.

## Materials and Methods

### Ethics Statements

Leukopac or blood from healthy human donors was obtained after written informed consent was provided and ethical approval granted from the Western Institutional Review Board.

All animals used in this study were housed and maintained at Genentech in accordance with American Association of Laboratory Animal Care guidelines. All experimental studies were conducted under protocols (#06-1535, #06-0967B and all subletters) approved by the Institutional Animal Care and Use Committee of Genentech Lab Animal Research in an AAALACi-accredited facility in accordance with the Guide for the Care and Use of Laboratory Animals and applicable laws and regulations.

### Reagents

A fully humanized anti-LT-α human IgG1 mAb, designated MLTA3698A, was derived from a mouse anti-human LT-α hybridoma mAb. The LT-α-specific Fc-mutant antibody (designated anti-LT-α-FcMT) that abolishes FcγR binding was made as previously described [10]. Human LT-βR.Ig, TNFRII.Ig, CTLA-4.Ig and isotype-IgG1 have been previously described [10], [14].

### Surface plasmon resonance analysis by BIACORE™

Surface plasmon resonance measurements on a Biacore 3000 instrument was used to characterize the interaction of LT-α3 or LT-α1β2 with anti-LT-α MLTA3698A. For IgG kinetic measurements, amine chemistry was used to covalently immobilize 8,000 RU of goat F(ab′)_2_ anti-human Fc polyclonal antibody (Jackson ImmunoResearch Laboratories) to 4 flow cells of a CM5 sensor chip. After blocking unreacted sites with 1 M ethanolamine-HCl pH 8, anti-LT-α MLTA3698A IgG was captured (100-500 RU) on the anti-Fc surface by injection of 7 µL of 0.5 µg/mL antibody at a flow rate of 10 µL/min. Solutions of recombinant LT-α3 (0, 50, and 100 nM) or LT-α1β2 (R&D Systems; 0, 200, 400, and 800 nM) were injected over the captured antibody and reference flow cells. Injections of 60 µL were used and dissociation was monitored for 20 minutes. Regeneration between samples was done using 80 µL injections of 10 mM glycine, pH 1.5. Data were analyzed according to a 1∶1 binding model, after subtraction of any reference cell signal, using BIAEval 3.1 to calculate kinetics constants.

### Binding and competitive blocking ELISA

Human LT-α1β2 and LT-α3 ELISA binding assays were performed by coating microtiter wells overnight with 0.5–1 µg/ml human LT-α1β2 or LT-α3 (R&D Systems), blocking with PBS containing 5 mg/ml bovine serum albumin, followed by incubation with purified or biotinylated anti-LT-α mAb, or LT-βR or TNFRII IgG fusion proteins. Detection was with streptavidin-horseradish peroxidase (SA-HRP) (Sigma) or horseradish peroxidase conjugated goat F(ab′)_2_ anti-human Fc polyclonal antibody (Jackson ImmunoResearch Laboratories) followed by tetramethylbenzidine. After the reaction was stopped with 1 M phosphoric acid, absorbances were read at 450 nm with a reference wavelength of 650 nm.

For competitive blocking ELISAs, recombinant LT-α3 or LT-α1β2 was labeled with biotin (Pierce, Thermo Scientific). Recombinant TNFRII.Ig and LT-βR.Ig were labeled using SULFO-TAG NHS-ester (Meso Scale Discovery). Test molecules were serially diluted, then biotinylated LT-α3 or LT-α1β2 added and incubated for two hr. Test molecule/biotinylated anti-LT-α mixtures were then added to streptavidin coated 96-well microtiter plates and allowed to bind for 30 min. After washing, SULFO-TAG TNFRII.Ig or LT-βR.Ig protein was added to the TNFRII.Ig or anti-LT-α/LT-α3 or LT-βR or anti-LT-α/LT-α1β2 binding pair titrations, respectively, and allowed to bind for 30 min. After washing and addition of Read Buffer T (Meso Scale Discovery), plates were immediately read on an MA6000 SECTOR™ Imager (Meso Scale Discovery).

### ADCC

ADCC was performed as previously described [10]. Briefly, NK cells were isolated from human PBMC using human NK Cell Isolation Kit (Miltenyi Biotec). Target cells were either 293-hLT-αβ cells (Young 2010) or CD4^+^ T cells purified from PBMC (CD4^+^ T Cell Isolation Kit, Miltenyi Biotec) stimulated *in vitro* for two days on wells precoated with 5 µg/ml anti-CD3 mAb (BD Biosciences) and in culture medium supplemented with 2 µg/ml anti-CD28 mAb (BD Biosciences). In duplicates, 10,000 target cells were pre-incubated with serially diluted mAbs for 30 min. 50,000 NK cells (E∶T ratio of 5∶1) were added and incubated for 4 hr at 37°C. Cell lysis was determined using Cytotoxicity Detection Kit (LDH) (Roche) and percentage cytotoxicity calculated.

### FACS and immune cell *in vitro* stimulation

Antibodies used for staining were as follows: FITC, PE or PerCP-conjugated anti-human CD4, CD8, CD19, CD25, CD45RO, CD56, CD69 and HLA-DR were purchased from BD Biosciences. Staining for surface LT-α1β2 was performed using anti-LT-α MLTA3698A or LT.3F12 mAb [15], anti-LT-α-FcMT or LT-βR.Ig [16]; all were Alexa-647-conjugated using Alexa Fluor 647 Protein Labeling Kit (Invitrogen). For detection of surface LT-α1β2 in animals treated with anti-LT-α mAb, staining was performed using the non-cross blocking anti-LT-α antibody LT.3F12 mAb which was shown to co-stain in the presence of MLTA3698A [15]. Leukopacs or blood from healthy donors was obtained after informed consent was provided and ethical approval granted from the Western Institutional Review Board. To track transferred human donor cells in the HuSCID model of GVHD and monitor cell expansion, donor cells were labeled with carboxyfluorescein succinimidyl ester (CFSE) cell tracer, using standard labeling procedures. Samples were acquired on a FACSCalibur flow cytometer using CellQuest Pro v5.1.1 software (Tree Star, Inc.). For determination of absolute cell numbers, CaliBRITE APC Beads (BD Biosciences) were added before analyzing samples by flow cytometry, and total cell numbers were determined according to manufacturer's instructions. For T cell activation, human PBMCs were cultured in complete DMEM media (DMEM supplemented with 10% FBS, 2 mM glutamine, 2 µM 2-ME, 1 mM sodium pyruvate, 100 U/ml penicillin and 100 µg/ml streptomycin) in presence of 5 µg/ml anti-CD3 mAb and 2 µg/ml anti-CD28 mAb for 2 days. For B cell activation, PBMCs were cultured in complete DMEM media supplemented with 100 ng/ml BAFF (R&D Systems) on microtiter plates precoated with 1 µg/ml anti-IgM (Jackson ImmunoResearch) for 2 days. For monocyte activation, CD14^+^ cells were isolated from peripheral blood using MACS separation and cultured in complete DMEM media supplemented with 1 µg/ml LPS (InvivoGen) for 2 days. LT expression on all *in vitro* stimulated human cells was detected using LT.3F12 mAb.

### Hu-SCID mouse model of GVHD

Leukopacs from normal human donors were obtained from Blood Centers of the Pacific (San Francisco, CA), and PBMCs were isolated using standard methodologies. CFSE-labeled human PBMCs were resuspended at a concentration of 50×10^6^ cells in 30 µL phosphate-buffered saline (PBS) and kept on ice prior to the intrasplenic injection procedure. SCID/beige mice were purchased from Charles River Laboratories (Hollister, CA) and housed and maintained at Genentech in accordance with American Association of Laboratory Animal Care guidelines. All experimental studies were conducted under the approval of the Institutional Animal Care and Use Committees of Genentech Lab Animal Research. Twenty female 5–6 week old female SCID/beige mice were sublethally irradiated with 350 rads using a Cesium-137 source. Polymyxin B (110 mg/L) and neomycin (1.1 g/L) were added to the drinking water for 7 days after irradiation. Four hours after irradiation, the left flank of each mouse was shaved and prepped with Betadine® (Purdue Pharma; Stamford, CT) and 70% alcohol. The operation was performed under anesthesia using aseptic surgical procedures. A 1-cm skin incision was made just below the costal border, followed by an incision of the abdominal wall and the peritoneum. The spleen was carefully exposed and injected with 50×10^6^ CFSE-labeled human PBMCs in 30 µL PBS. The incisions were closed in the muscular layer and the skin with 5-O Vicryl® sutures (Ethicon; Somerville, NJ) and surgical staples, respectively. For lymphoid cell phenotyping and determining expansion of human donor cells based on CFSE profile, mice were sacrificed at days 1, 2, 3, and 4 post-transplantation, and spleens harvested. For survival studies, each animal received 50×10^6^ unlabeled human PBMCs via i.v. tail vein injection in 100 µL sterile PBS, four hours after irradiation and dosing with experimental or control test articles. Isotype control mAb, anti-LT-α, anti-LT-α-FcMT or CTLA-4.Ig, were administered as i.p. injections at a dose of 12 mg/kg in 100 µL of saline, and treatment was continued with twice-weekly injections. Survival of mice was monitored through day 28 and data were plotted as Kaplan-Meier survival curves. Drug bioavailability was confirmed by ELISA, and average serum drug concentrations were 100–200 µg/ml at all time points examined for all treatments.

### Statistical analyses

Statistics were calculated using JMP version 6.0.2 software (SAS Institute). We made comparisons for each pair with Student's *t* test; we made multiple comparisons with a single control with Dunnett's test; we compared survival using log-rank test. *P* values<0.05 were considered significant. EC_50_ curves and values were plotted and calculated using KaleidaGraph version 3.6 software (Synergy Software) using a 4-parameter curve fit formula: M1+(M2−M1)/(1+(M0/M4)∧M3); M1 = minimum of curve, M2 = maximum of curve, M3 = 1, M4 = estimated EC_50_.

## Results

### LT-α is expressed by activated lymphocytes

**Figure 1:**
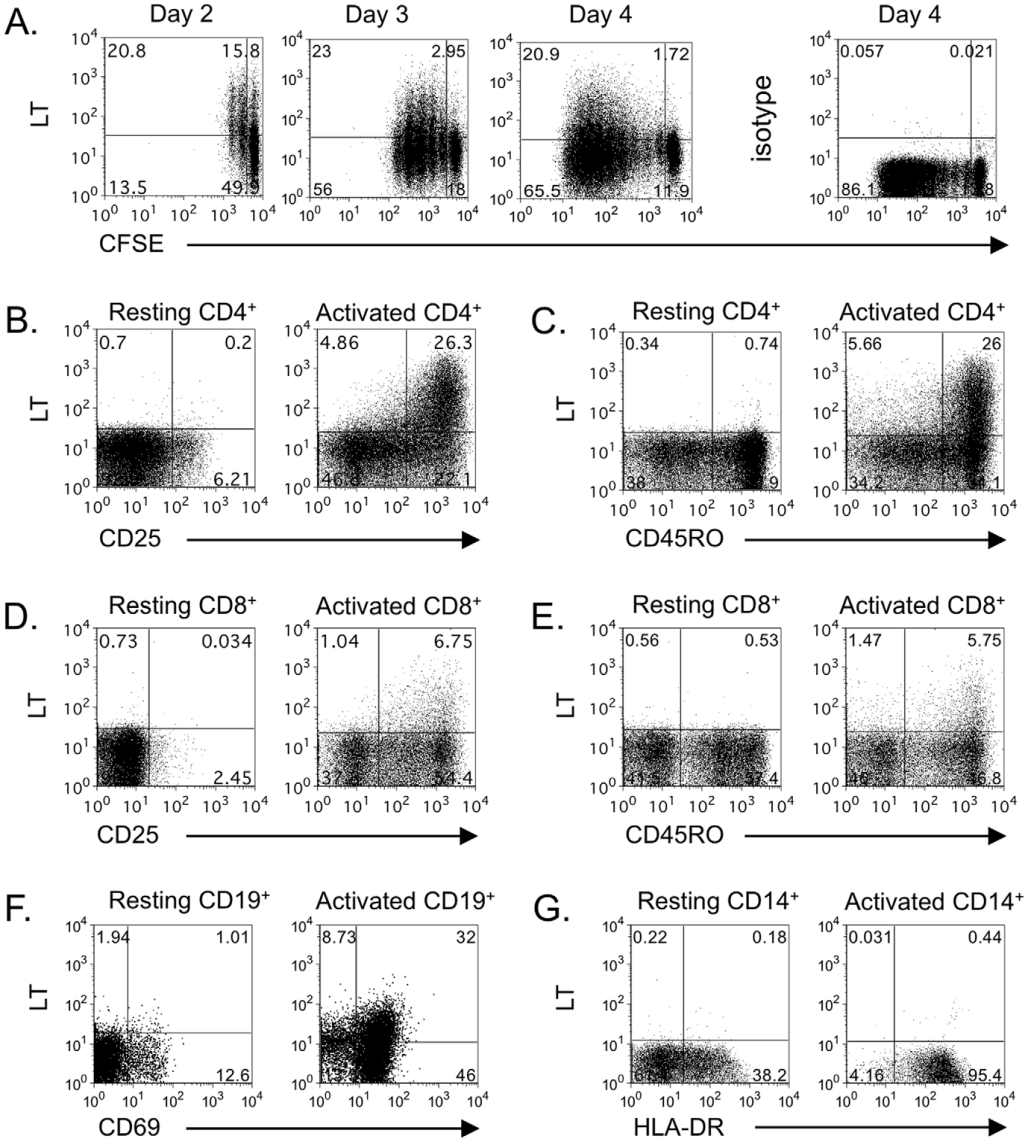
LT expression on human immune cell populations. Human CD4^+^ T cell expression of LT. **A**. Sorted CD4^+^ T cells were labeled with CFSE and proliferation and LT expression was monitored at indicated time points following stimulation with anti-CD3 and anti-CD28. **B**, **C**. Co-expression of LT with T cell activation markers CD25 (**B**) or CD45RO (**C**) on CD4^+^ gated cells. **D**, **E**. CD8^+^ T cell expression of LT. Co-expression of LT with CD25 (**D**) or CD45RO (**E**) on CD8^+^ gated cells. For CD4^+^ and CD8^+^ T cell activation, PBMCs were stimulated with anti-CD3 and anti-CD28 mAbs for 2 days. **F**. Human B cell expression of LT. B cells were stimulated with anti-IgM and BAFF for two days, then LT expression determined on CD19^+^ B cells with CD69 as a marker for activation. **G**. Human monocyte expression of LT. CD14^+^ monocytes were stimulated with LPS, with activation status assessed on the basis of HLA-DR up-regulation.

As activated T and B cells are key mediators of GVHD, we determined if these subsets express surface LT-α in a xenogeneic Hu-SCID model of GVHD. We initially assessed surface LT-α expression on lymphocytic populations isolated from human PBMC and activated *in vitro* (Figure 1). Surface LT-α is expressed rapidly on T cells after activation [10], [11] and is maintained throughout cell-division in bulk CD4^+^ T cell cultures (Figure 1A). Activated CD4^+^ T cells co-expressed LT-α1β2 with other T cell activation markers CD25 and CD45RO (Figure 1B, C). It has been previously reported that Th1, Th0 and Th17 subsets express the highest level of LT-α, compared to Th2 that only express low transient levels [10], [11]. LT-α1β2 was also expressed on the surface of activated memory CD8^+^ T cells; however, expression was lower than that observed on CD4^+^ T cells (Figure 1 D, E). On non-T cell populations, LT-α1β2 was expressed on B cells stimulated with anti-IgM and BAFF (Figure 1F), but not on LPS-stimulated monocytes (Figure 1G). These data confirm and extend previous studies reporting on the expression pattern of LT-α1β2 on immune cell subsets [10], [11], [12], [13].

**Figure 2:**
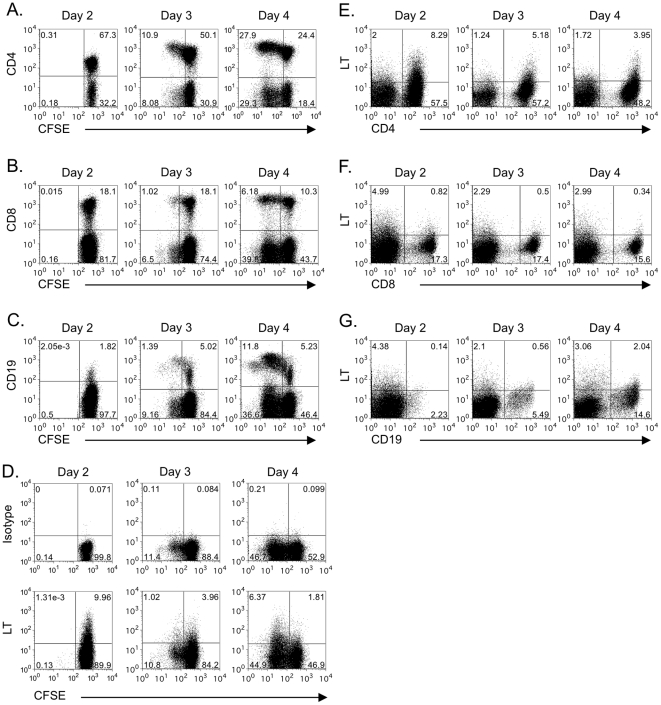
Expansion and expression of surface LT on human lymphocytes following transfer into SCID animals. CFSE-labeled human PBMCs were transferred into SCID mice via intrasplenic injection, then proliferation (**A–C**) or surface LT expression (**D–G**) was determined by flow cytometry. At indicated time points after transfer, spleen cells were harvested and human lymphocyte populations were identified on the basis of CFSE and specific cell marker staining. Proliferation of CD4^+^ T cells (**A**), CD8^+^ T cells (**B**) and CD19^+^ B cells (**C**). Surface LT expression on CFSE-labeled bulk transferred human PBMCs (**D**), or CFSE^+^ gated CD4^+^ T cells (**E**), CD8^+^ T cells (**F**) and CD19^+^ B cells (**G**). In each experiment, 2–3 spleens were pooled to provide sufficient cell numbers for data collection. Data are representative of staining for 1 pool out of 3 per experiment. A minimum of 3 experiments were performed for each cell type.

To determine whether LT expression is induced *in vivo* on immune cell subsets in the xenogeneic Hu-SCID model of GVHD [17], unfractionated CFSE-labeled human PBMCs were transferred intrasplenically into SCID recipient mice, and cellular expression of LT examined daily by flow cytometry. We first examined the presence and expansion of immune cell subsets in the early days of engraftment. Cells were readily detected 2 days post-transfer, and T and B cells were proliferating by day 3, as determined by CFSE dilution (Figure 2A–C). On day 2, LT-α was already expressed on 10% of total human donor lymphocytes (Figure 2D) and the majority of these were CD4^+^ T cells (Figure 2E–G). We were unable to detect any significant levels of soluble serum LT-α3 in these animals. As cells proliferated, CD4^+^ and CD8^+^ T cells maintained surface LT-α expression while surface LT-α was upregulated on B cells (Figure 2E–G, Figure S1). Overall, human PBMCs express surface LT-α soon after transfer and activation in SCID mice.

### Generation and properties of humanized depleting anti-LT-α mAb

We have previously shown that depletion of LT-expressing cells ameliorated disease in a number of inflammatory and autoimmune disease models using a mAb directed against mouse LT-α [10]. To determine whether depletion of LT-expressing cells in the xenogeneic Hu-SCID model of GVHD would also achieve therapeutic benefit, we generated a fully humanized anti-LT-α antibody, designated MLTA3698A, selected for its ability to bind LT-α in its different trimeric forms and to mediate depletion of LT-α-expressing cells.

**Figure 3:**
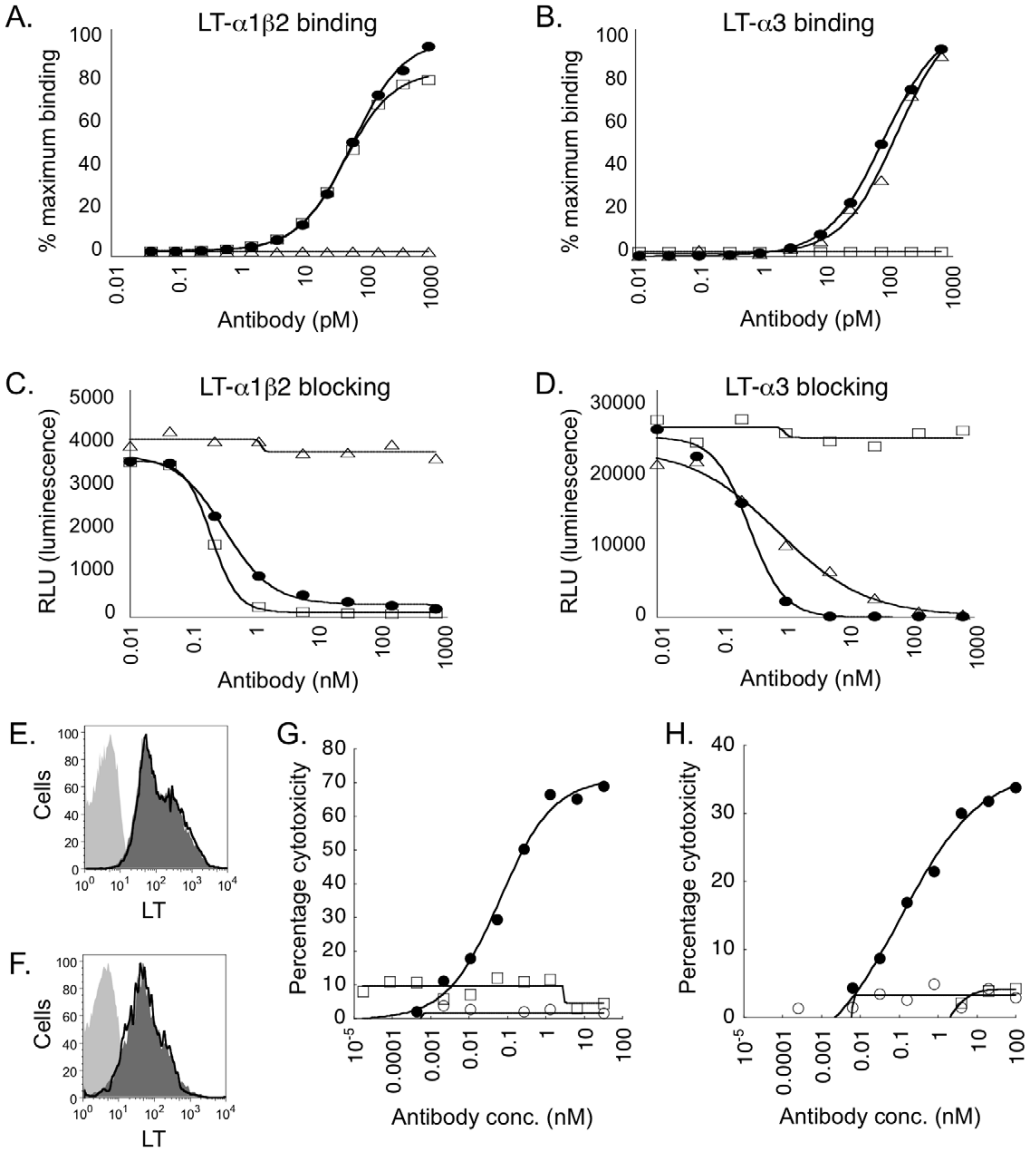
Binding, blocking and depleting properties of anti-LT-α mAb. **A**. Anti-LT-α MLTA3698A (filled circles) LT-βR.Ig (open squares) and TNFRII.Ig (open triangles) binding to LT-α1β2 in ELISA binding assays. **B**. ELISA binding to LT-α3. **C**. Blockade of LT-βR.Fc binding to LT-α1β2 in ELISA competition assays with anti-LT-α MLTA3698A (filled circles), LT-βR.Ig (open squares) or TNFRII.Ig (open triangles). **D**. Blockade of TNFRII.Fc binding to LT-α3 in ELISA competition assays. ADCC activity of anti-LT-α mAb against LT-expressing cells. LT expression on 293-hLT-αβ cells (**E**) and activated human CD4^+^ T cells (**F**) was detected using anti-LT-α MLTA3698A (dark gray shaded histograms), anti-LT-α-FcMT mAb (solid line). Isotype control antibody staining is indicated by light-shaded histograms. Data are representative of at least five experiments. ADCC activity against 293-hLT-αβ (**G**) or activated CD4^+^ T cell (**H**) targets mediated by MLTA3698A (filled circles) or anti-LT-α-FcMT(open circles). Isotype control mAb is indicated by open squares. Data are representative of three experiments.

Anti-LT-α MLTA3698A was derived from a mouse anti-human LT-α hybridoma mAb, fully humanized after fusing respective V_L_ and V_H_ domains to the constant domains of human kappa L chain and human IgG1 H chain, and then affinity matured [18], [19], [20]. The ability of MLTA3698A to bind to LT-α3 and LT-α1β2 was confirmed by surface plasmon resonance. Kinetic affinity analysis showed that MLTA3698A bound to LT-α3 and LT-α1β2 with affinity constants of 0.4 nM (ka = 1.9×10^5^ M^−1^ s^−1^ and kd = 7.9×10^−5^ s^−1^) and 8.7 nM (ka = 2.7×10^4^ M^−1^ s^−1^ and kd = 2.4×10^−4^ s^−1^), respectively. In ELISA assays, MLTA3698A bound to LT-α1β2 with an EC_50_ value of 42 pM, comparable to that of LT-βR.Ig (EC_50_ = 48 pM), and bound LT-α3 with an EC_50_ of 69 pM, similar to TNFRII.Ig (EC_50_ = 133 pM) (Figure 3A, B). As controls, LT-βR.Ig did not bind LT-α3, and TNFRII.Ig did not bind LT-α1β2, as expected. Furthermore, MLTA3698A blocked binding of LT-βR.Ig to LT-α1β2 and TNFRII.Ig to LT-α3 in a competitive dose-dependent manner (Figure 3C, 3D). MLTA3698A blocked LT-βR.Ig binding with an IC_50_ value of 0.31 nM, comparable to LT-βR.Ig's ability to block itself (IC_50_ = 0.1 nM). Similarly, anti-LT-α blocked TNFRII.Ig binding with an IC_50_ value of 0.29 nM, comparable to TNFRII.Ig's ability to block itself (IC_50_ = 0.83 nM).

Critical for the mechanism of action of anti-LT-α in the GVHD model, we verified that MLTA3698A was functionally capable of depleting LT-α-expressing cells in ADCC assays. To control for FcγR-mediated depletion, a Fc mutant version of the wild-type anti-LT-α MLTA3698A (anti-LT-α-FcMT) was generated that lacks the ability to bind Fcγ receptors [10], [21]. Both anti-LT-α and anti-LT-α-FcMT mAbs bound surface LT-α1β2 comparably as determined by flow cytometry on either 293 cells stably transfected with LT-α and LT-β (293-hLT-αβ cells) or activated human CD4^+^ T cells (Figure 3E, F). In *in vitro* ADCC assays performed using 293-hLT-αβ target cells, anti-LT-α elicited dose-dependent cytotoxicity whereas the effectorless anti-LT-α-FcMT mAb was unable to mediate cell killing (Figure 3G). Anti-LT-α had no effect on non-transfected 293 cells alone (data not shown). To extend this observation to primary immune cells, activated human CD4^+^ T cells were used as targets and were similarly killed in a dose-dependent manner with anti-LT-α but not with anti-LT-α-FcMT (Figure 3H). Thus, MLTA3698A is a depleting mAb that specifically binds soluble and surface expressed LT-α and has the capacity to mediate ADCC via Fcγ receptor-dependent mechanisms.

### Anti-LT-α mAb depletes LT-α-expressing cells *in vivo*

**Figure 4:**
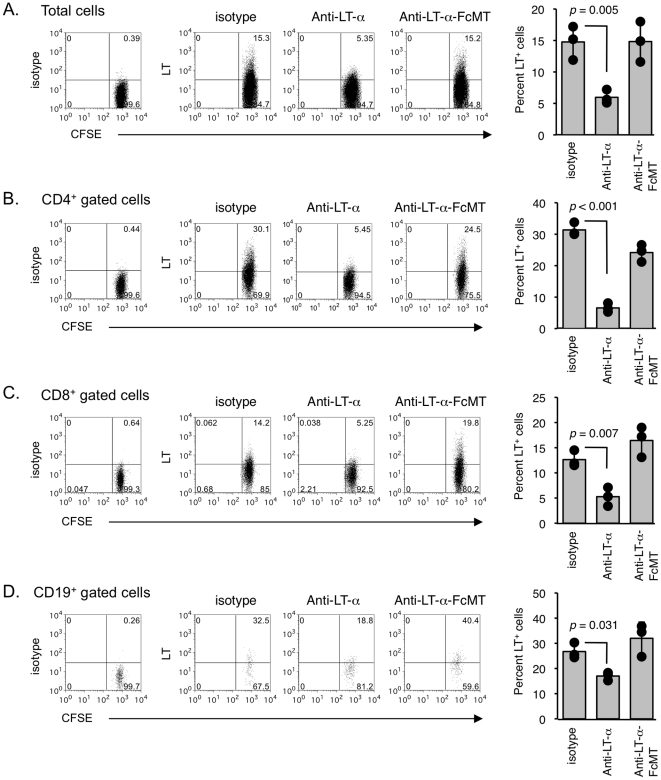
LT-α-specific mAb depletes LT-expressing human lymphocytes in Hu-SCID GVHD model. Spleens were harvested from SCID mice two days following intrasplenic injection of human PBMCs, after one day treatment with anti-LT-α MLTA3698A, anti-LT-α-FcMT or isotype control mAb. **A**. LT staining on total CFSE-labeled transferred cells and enumeration of percentage of total human cells expressing surface LT. **B**–**D**. LT expression and quantitation of mAb treatment effects on specific human lymphocyte populations. Spleen cells were gated on CD4^+^ cells (**B**), CD8^+^ cells (**C**) or CD19^+^ cells (**D**). In each experiment, 2–3 spleens were pooled to provide sufficient cell numbers for data collection. Data show staining for 1 pool out of 3 per experiment, and are representative of a minimum of 2 experiments for each cell type.

We then determined whether the MLTA3698A mAb could deplete LT-α-expressing human immune cells *in vivo* using the Hu-SCID GVHD model. SCID mice engrafted with human CFSE-labeled PBMC by intrasplenic injection were treated one day post-transfer with either wild-type anti-LT-α and anti-LT-α-FcMT mAb or human IgG1 isotype control. Spleens were harvested on the following day (day 2 post-transfer) and the percentage of total transferred cells expressing LT determined by flow cytometry (Figure 4). Mice treated with anti-LT-α had a 3-fold decrease in total LT-α-expressing cells compared to anti-LT-α-FcMT and isotype control mAb treated animals (Figure 4A). Further characterization of CD4^+^, CD8^+^, and CD19^+^ immune cell subsets showed that the decrease observed with anti-LT-α was reflected in all these subsets (Figure 4B–D). After one day of treatment with anti-LT-α, the frequencies of CD4^+^ T cells, CD8^+^ T cells and B cells were decreased 80%, 60% and 35% respectively. The low frequency of CD4^+^ T cells was also reflected in diminished expansion of CD4^+^ T cells, as evidenced by the paucity of CFSE-diluted proliferating cells, in the anti-LT-α treatment group at day 4 post-transfer (Figure S2). Cumulatively, anti-LT-α-mediated depletion was demonstrated to require FcγR-dependent pathways and target T cells and B cells expressing surface LT-α *in vivo*.

### A depleting anti-LT-α mAb inhibits human lymphocyte-mediated GVHD

**Figure 5:**
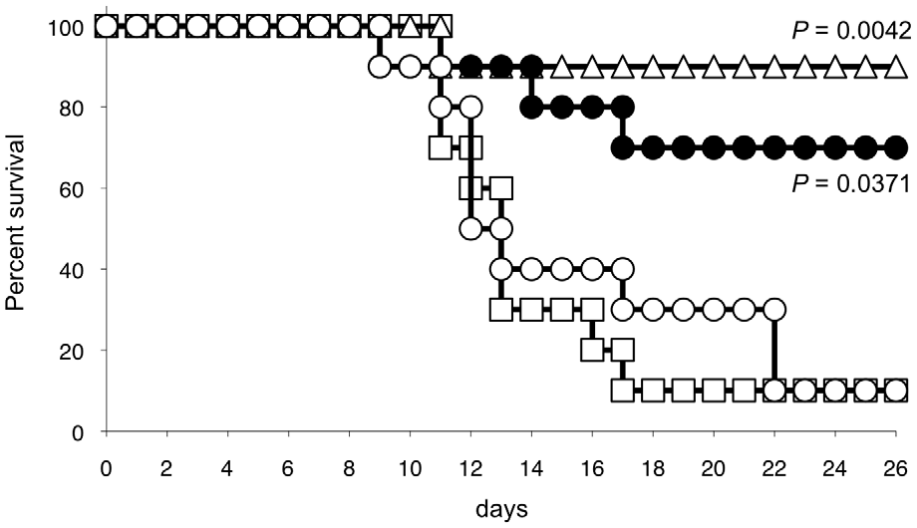
Efficacy of anti-LT-α mAb in Hu-SCID model of GVHD. SCID mice were sublethally irradiated, randomized, then immediately treated with anti-LT-α MLTA3698A (filled circles), anti-LT-α-FcMT (open circles), CTLA-4.Ig (open triangles) or isotype control Ab (open squares). 4 hr after initial treatment, human PBMC were transferred via i.v. injection. Treatment was continued with twice-weekly dosing throughout the study. Two independent studies were performed with similar results.

Since anti-LT-α mAb anti-LT-α depleted LT-α-expressing immune cells *in vivo*, we tested whether this mechanism would provide efficacy in a standard survival study in this Hu-SCID GVHD model. SCID mice were engrafted intravenously with unlabeled human PBMC, and cohorts treated twice-weekly with i.p. injections of isotype control, CTLA4.Ig therapeutic positive control or experimental anti-LT-α mAbs, beginning on the day of transfer, for 28 days. Isotype control-treated SCID mice succumbed to fatal GVHD-like syndrome within 17 days with a mean (± SEM) survival of 14.5±1.6 days as expected (Figure 5). Mice treated with anti-LT-α had significantly prolonged survival (mean survival of 23.6±2.3 days) compared to the isotype control group (log rank, *p*<0.01). The beneficial survival effect of anti-LT-α was similar to that observed with CTLA-4.Ig treatment, where mean survival was 26.3±1.7 days, resulting in 12 days prolonged survival compared to the isotype group (log rank, *p*<0.001). Anti-LT-α-FcMT did not delay mortality, with mean survival of 15.8±2.0 days, indicating that FcγR-mediated killing of LT-α-expressing cells was a requisite part of the anti-LT-α-driven survival.

## Discussion

We report that targeting subsets of activated immune cells expressing surface LT-α for depletion using an humanized anti-LT-α specific depleting antibody resulted in prolonged survival in xenogeneic Hu-SCID model of GVHD. LT-α exists as a soluble homotrimer or at the cell surface complexed in a heterotrimer with LT-β. Surface expression of LT-α1β2 is transiently upregulated on subsets of activated T and B cells (shown here and [10]). We previously demonstrated that a depleting mouse anti-LT-α mAb provided therapeutic efficacy in a number of mouse inflammatory and autoimmune models. In a mouse model of arthritis, mouse anti-LT-α efficacy was attributed to depletion of Th1 and Th17 cells, T cell subsets that express LT-α and are the pathogenic drivers of disease. Conversely, Th2 cells do not express abundant surface LT-α and, not surprisingly, anti-LT-α had no effect in a Th2-driven model of asthma. We extend these findings to the human immune system by using the Hu-SCID GVHD model. Analyses of LT expression on specific immune cell subsets revealed that LT expression was induced on human CD4^+^ and CD8^+^ T cells and B cells soon after transplantation into recipient mice, and that treatment with fully humanized depleting anti-LT-α mAb MLTA3698A significantly reduced the frequency of these activated effector cells within one day of administration. The elimination of these potentially pathogenic mediators results in prolonged survival of transplant recipients.

In humans, allogeneic hematopoietic stem cell transplantation is an effective therapy for a number of hematological conditions. While conditioning regimens that include radiation and chemotherapy enable engraftment, a significant number of patients develop donor T cell-mediated GVHD, a major cause of morbidity and mortality. It is well documented that donor CD4^+^ and CD8^+^ T cells are the primary mediators of GVHD, resulting from their activation in response to alloantigen presentation by host APC. B cells may also play a role in GVHD pathology, with incidence of acute GVHD mortality correlated with higher numbers of B cells in the apheresis product [22], higher BAFF levels and increased numbers of activated memory B cells associated with chronic GVHD [7]. As such, a number of therapeutic approaches employing mAbs directed against cellular surface markers have been tested in the clinic as salvage treatments in steroid refractory/non-responsive GVHD. However, clinical efficacy has been limited by increased incidence of graft failure, leukemia relapse, and susceptibility to post-transplant infection [3], [8]. This may be due to the nonspecific nature of existing mAbs targeting T cells. For example, OKT3 and visilizumab (CD3) broadly target all T cells in a “pan” manner, not discriminating between T cells that are alloreactive and those that are nonalloreactive [23], [24]. Alemtuzumab is more promiscuous, targeting CD52, an antigen expressed not only by T cells, but also by B cells, NK cells, monocytes, macrophages, dendritic cells and eosinophils [25]. Daclizumab, basiliximab and denileukin-difitox target CD25 expressed on activated T cells, and to a lesser extent on resting T cells. However, CD25 is also expressed by CD4^+^ regulatory T cells, which are important for inducing tolerance that preserves the graft-versus-leukemia effect following transplantation [26]. Alefacept recognizes CD2, and selectively targets effector memory T cells, which may result in impairment of memory responses against infections [27], [28]. AXL-CBL is a mAb that attempts to eliminate those immune cells directly involved in GVHD pathology, targeting CD147 that is highly expressed on activated T and B cells as well as monocytes, macrophages and dendritic cells [29], [30].

The success of these various therapeutic approaches as salvage treatments in GVHD, despite the aforementioned limitations associated with side effects, primarily susceptibility to infection, prompted us to search for cell surface molecules expressed on those cell populations involved in GVHD pathogenesis that are amenable for targeted depletion. As an additional criterion, we sought to identify surface molecules upregulated upon activation, rather than those constitutively expressed, as this would allow for targeting of the relevant cell types actively involved in mediating disease, rather than “pan” depletion of all cells including those that are naïve or quiescent. The depleting anti-LT-α mAb described in this report eliminates those cells expressing surface LTα1β2. LT expression on human T cells and B cells is induced soon after transfer into SCID recipient mice, and expression is maintained predominantly on activated, proliferating cells.

As LTα1β2 appears to selectively and transiently mark activated T and B lymphocytes, we tested whether anti-human LTα mAb had therapeutic impact in the chimeric Hu-SCID model of GVHD. This model of GVHD requires human CD4^+^ T cells [17] and allows for ADCC lysis of human target cells *in vivo* due to the presence of functional human NK cells [31]. Mice treated with depleting anti-LT-α had significantly prolonged survival compared isotype control Ab-treated animals. No protective effect was seen with a Fc-effectorless mutant anti-LTα Ab, demonstrating that efficacy could be attributed to depletion of targeted cells. The depleting mechanism of action was supported by significant reductions in frequencies of LT-expressing CD4^+^ and CD8^+^ T cells as well as B cells as early as two days after treatment. The physiological consequences of depletion of CD8^+^ T cells and B cells on disease outcome in the Hu-SCID model are unclear as others have shown that depletion of these cells did not prevent or delay development of GVHD in a similar xenogeneic model [32]. Thus, while the Hu-SCID model may have pathological differences from human disease, anti-LT-α mAb may have the potential to remove other immune components contributing to GVHD in human patients.

Our data provide compelling evidence that targeted depletion of recently activated, pathogenic immune effector cells with anti-LT-α mAb has promise as a therapeutic strategy for prevention of GVHD. This data complements our studies using mouse surrogate reagents to deplete LT-α-expressing cells and achieve therapeutic benefit in various animal models of autoimmune disease, including RA and EAE [10]. Anti-LT-α mAb eliminated alloreactive GVHD mediators by depletion in HuSCID mice, and thus represents a potential new therapy for the treatment of GVHD. Anti-LT-α may also have wider applications in treatment of other T cell- and/or B cell-mediated diseases, including autoimmune disease.

## Supporting Information

Figure S1 Expression of surface LT on human lymphocyte populations following transfer into SCID animals. Proliferation and surface LT expression on CFSE-labeled human CD4^+^ T cells (A), CD8^+^ T cells (B), or CD19^+^ B cells (C). CFSE-labeled human PBMCs were transferred into SCID mice via intrasplenic injection. At indicated time points after transfer, spleen cells were harvested then LT expression on proliferating cells, assessed on the basis of CFSE dilution, was determined by flow cytometry. Staining for specific cell markers was used to identify immune cell populations in CFSE^+^ gated cells. In each experiment, 2–3 spleens were pooled to provide sufficient cell numbers for data collection. Data are representative of staining for 1 pool out of 3 per experiment. A minimum of 3 experiments were performed for each cell type.
(TIF)

Figure S2 Anti-LT-α mAb reduces frequency of LT-expressing proliferating human CD4^+^ T cells in Hu-SCID GVHD model. Spleens were harvested from SCID mice four days following intrasplenic injection of human PBMCs, after three days treatment with anti-LT-α MLTA3698A, anti-LT-α-FcMT or isotype control mAb. Cells were gated for CD4^+^ T cells, then LT expression was analyzed on CFSE-labeled transferred cells. Data are representative of three experiments.
(TIF)
