# Supplementary figures and images for: In Vivo Depletion of Lymphotoxin-Alpha Expressing Lymphocytes Inhibits Xenogeneic Graft-versus-Host-Disease
Source: PLoS One. 2012 Mar 12;7(3):e33106. doi: 10.1371/journal.pone.0033106 (PMC3299734; doi:10.1371/journal.pone.0033106)

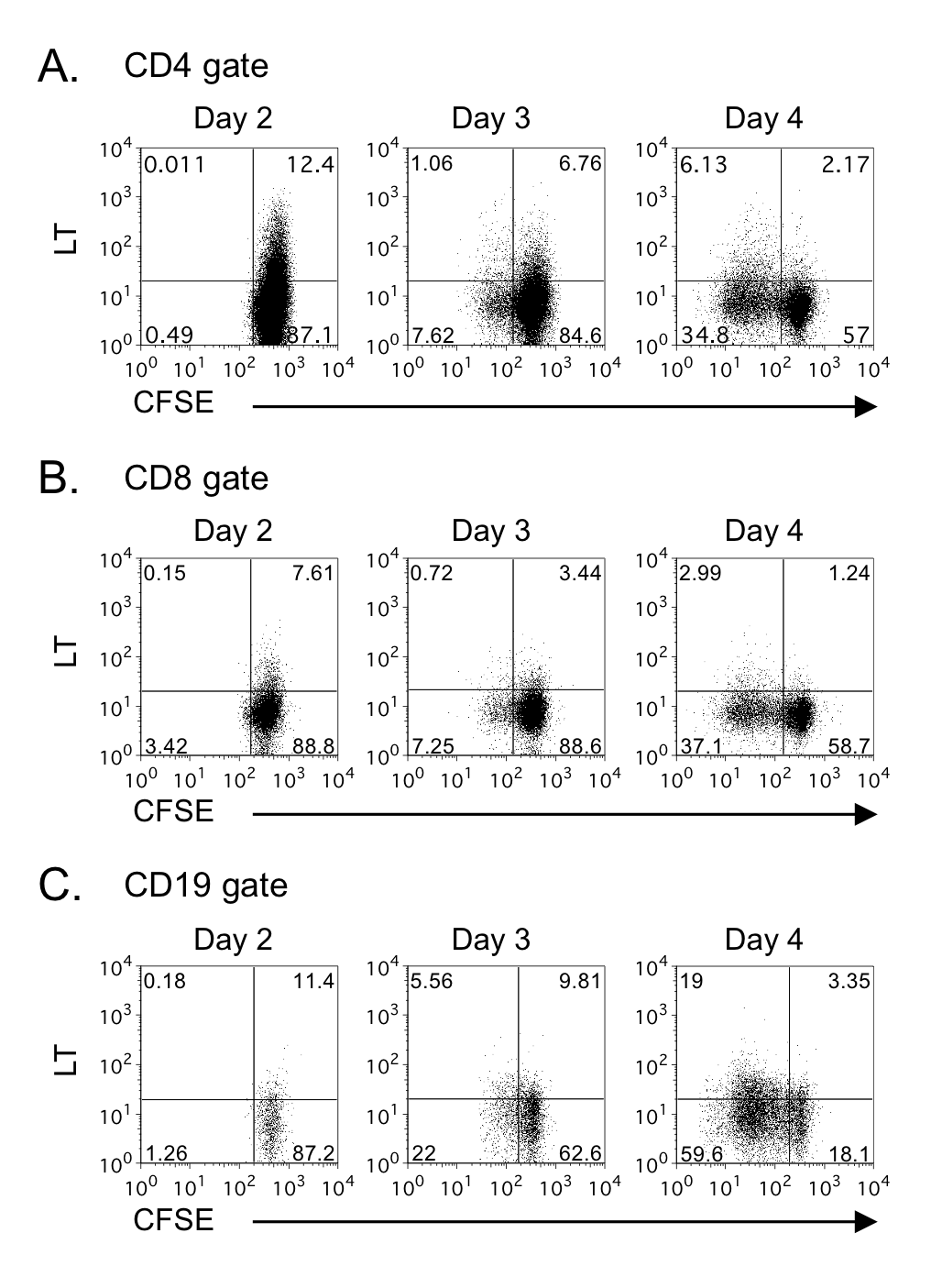

Supplement: Figure S1 — Expression of surface LT on human lymphocyte populations following transfer into SCID animals. Proliferation and surface LT expression on CFSE-labeled human CD4+ T cells (A), CD8+ T cells (B), or CD19+ B cells (C). CFSE-labeled human PBMCs were transferred into SCID mice via intrasplenic injection. At indicated time points after transfer, spleen cells were harvested then LT expression on proliferating cells, assessed on the basis of CFSE dilution, was determined by flow cytometry. Staining for specific cell markers was used to identify immune cell populations in CFSE+ gated cells. In each experiment, 2–3 spleens were pooled to provide sufficient cell numbers for data collection. Data are representative of staining for 1 pool out of 3 per experiment. A minimum of 3 experiments were performed for each cell type. (TIF) [file pone.0033106.s001.tif]

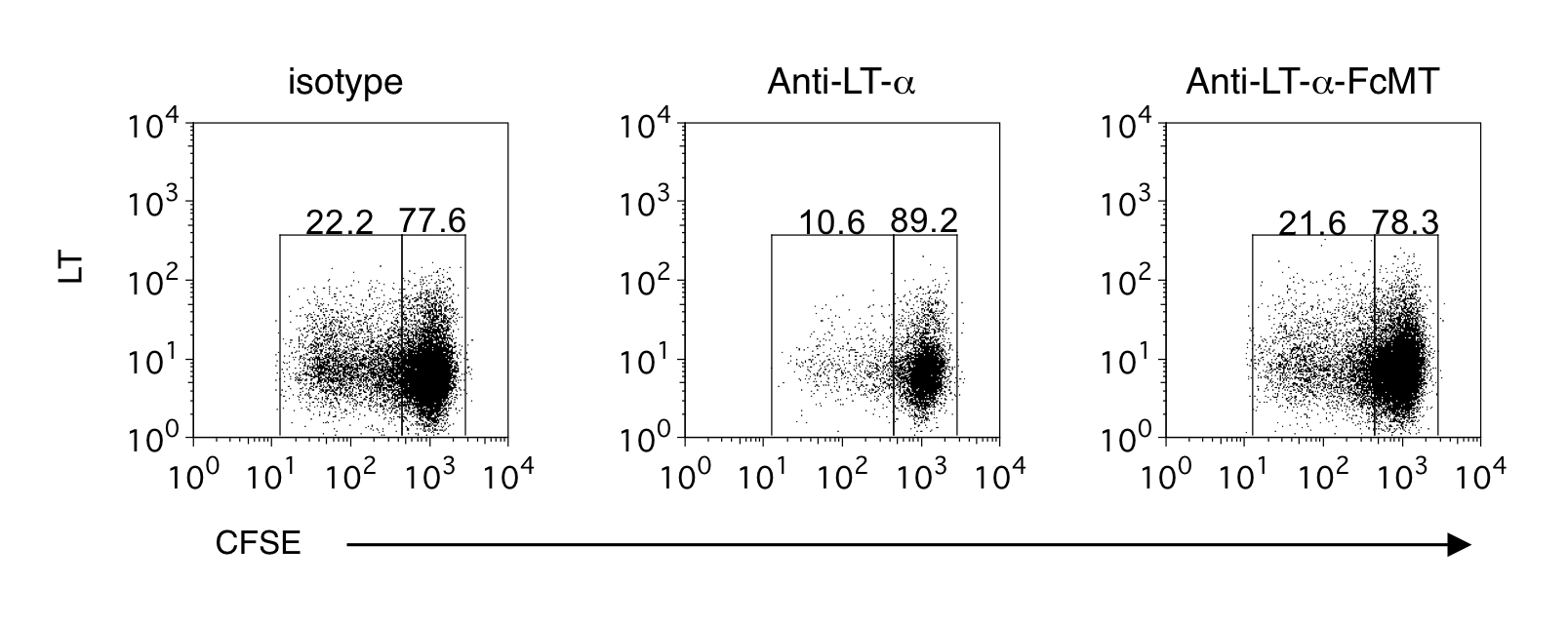

Supplement: Figure S2 — Anti-LT-α mAb reduces frequency of LT-expressing proliferating human CD4+ T cells in Hu-SCID GVHD model. Spleens were harvested from SCID mice four days following intrasplenic injection of human PBMCs, after three days treatment with anti-LT-α MLTA3698A, anti-LT-α-FcMT or isotype control mAb. Cells were gated for CD4+ T cells, then LT expression was analyzed on CFSE-labeled transferred cells. Data are representative of three experiments. (TIF) [file pone.0033106.s002.tif]
